# Factors influencing shared decision-making in long-term care facilities

**DOI:** 10.1186/s12877-023-04301-6

**Published:** 2023-09-19

**Authors:** Da Eun Kim, Min Jung Kim

**Affiliations:** 1 College of Nursing and Research Institute of Nursing Science, Kyungpook National University, Daegu, Republic of Korea; 2 Department of Nursing, Kyongbuk Science College, Gyeongsangbuk-do, Republic of Korea

**Keywords:** Shared decision-making, Person-centered care, Long-term care, Aged

## Abstract

**Background:**

Shared decision-making, a communicative process to reach decisions based on informed preferences, evidence, and co-created goals, improves care satisfaction and patients’ quality of life. However, shared decision-making has not been widely implemented in long-term care facilities, and few studies have examined how to promote the shared decision-making practice. This study aimed to identify the influencing factors of shared decision-making based on the Person-centered Practice Framework in long-term care facilities.

**Methods:**

A total of 300 staff (nursing staff, social workers, and personal care workers) in 13 Korean long-term care facilities participated in this study. Data from 280 respondents were finally analyzed, excluding respondents with missing values. Data were collected using structured questionnaires that included items on shared decision-making, personal factors (e.g., knowledge about dementia, person-centered care education, person-centered attitude, communication behavior, and job tenure), and care environment factors (e.g., person-centered climate, staffing level, effective staff relationships, supportive supervisors, and power-sharing). Multilevel linear regression analyses were performed using Mplus Version 8.8.

**Results:**

The mean shared decision-making score was 35.78 (range 8–45). Staff with experience of person-centered care education (β = 0.198, *p* = 0.034), a higher person-centered attitude score (β = 0.201, *p* = 0.007), and a higher communication behavior score (β = 0.242, *p* < 0.001) were more likely to report a higher shared decision-making score. In addition, staff who viewed their care environment as more person-centered were more likely to report a higher shared decision-making score (β = 0.416, *p <* 0.001).

**Conclusions:**

This study highlights that personal (e.g., person-centered care education, person-centered attitude, and communication behavior) and care environment (e.g., person-centered climate) factors could influence shared decision-making for long-term care residents. These findings could be foundational evidence for facilitating shared decision-making practice in long-term care settings.

**Supplementary Information:**

The online version contains supplementary material available at 10.1186/s12877-023-04301-6.

## Background

Shared decision-making (SDM) is an increasingly prominent approach in healthcare, focusing on the effective two-way exchange of information and perspectives between healthcare professionals and patients regarding various care options [1]. SDM is a collaborative approach involving the active participation of healthcare professionals and patients in all phases of the decision-making process with the aim of working together to determine the most suitable treatment option among the available choices [2]. It has gained increasing popularity in clinical practice due to the higher effectiveness of treatments when patients’ self-determination is acknowledged and care is provided in an ethical way [1]. According to a previous study, the active engagement of patients in treatment decisions had a positive impact on patient satisfaction [3], as well as improved their self-care and self-efficacy [4]. Moreover, for patients with chronic conditions, such engagement minimized disability and complications, ultimately enhancing their quality of life [5]. Notably, SDM has shown promising results in frail older adults requiring long-term healthcare, leading to appropriate medical decisions and better health outcomes [6].

There has been increasing discussion around the need to apply SDM in long-term care settings [7]. Because SDM is a process that highlights personal values and preferences to help individuals make the most appropriate decisions for themselves, it is a concept that coincides with person-centered care that aims to provide individualized care considering residents’ personal preferences, needs, strengths, and functional levels [8]. In long-term care facilities, it is crucial to recognize that residents possess unique sets of values and increase the participation of residents and/or their families in the decision-making process regarding care. An intervention study conducted in nursing homes in Italy and the Netherlands demonstrated that SDM involving residents and families in the care planning process improves personalized care plans [9]. Furthermore, SDM can ensure the autonomy of long-term care residents [10] and increase the well-being of individuals with dementia and their families [11].

While SDM enables long-term care staff to focus on each resident`s unique needs and values, it is yet to be actively applied in long-term care facilities for older adults. One reason is the lack of knowledge and education among healthcare providers regarding SDM, thereby hindering its implementation [12]. Additionally, there is a tendency in the decision-making process to underestimate the level of engagement desired by patients [12]. Notably, in the case of older adults experiencing cognitive decline, which impairs their ability to express their needs [13], fostering open and proactive communication to strengthen the relationship between staff and residents becomes crucial for promoting SDM [7]. Furthermore, work environment factors, such as time constraints and ambiguity of job descriptions, have been shown to impede SDM [14]. Consequently, several factors contribute to the limited implementation of SDM in long-term care facilities. However, few studies have investigated the current status and influencing factors that promote SDM in long-term care facilities in South Korea.

With the rapid increase in Korea’s older population, the long-term care insurance system was established in 2008 as a social insurance to provide care for the older adults experiencing challenges in their daily lives and to relieve the care burden on their family caregivers. In Korea, the number of long-term care facilities more than tripled from approximately 1,700 at the end of 2008 to 6,334 at the end of 2021; there were 230,170 facility benefit recipients at the end of 2021 [15]. While the number of benefit recipients and institutions continues to grow, quality concerns such as a task-oriented approach, a lack of self-determination, and low-quality care persist. In an effort to address these quality issues, person-centered care models (e.g., Eden Alternative, Green House, Wellspring, and relationship-centered models) were introduced in long-term care facilities throughout the United States and the United Kingdom. Meanwhile, in Korea, no distinct person-centered care model has been devised to date, and most care facilities mainly provide provider-centered or physical demand-oriented care [16]. However, because some indicators related to SDM are included in nursing home accreditation, which is conducted every three years by the Korean government to evaluate care quality with the results being made available to consumers, long-term care facility staff strive to perform SDM to ensure recipients’ rights and provide individualized care [17]. These indicators include providing adequate information and ensuring choice, responding to residents’ or families’ opinions during the care process, and individualized care planning through comprehensive needs assessment. Long-term care residents, in particular, have complicated care needs, necessitating a multidisciplinary and collaborative approach to care [7]. Therefore, identifying the current status and influencing factors of performing SDM among Korean long-term care facility employees (e.g., nursing staff, social workers, and personal care workers) is essential.

Adopting a person-centered care approach focused on residents’ individual characteristics and ensuring their autonomy and self-determination is essential to ensure effective SDM [8, 18]. Therefore, it is beneficial to examine the factors that influence SDM using the principal person-centered care models. The Person-centered Practice Framework (PCPF) is a practical and systematic model that introduces four domains and relevant components to guide person-centeredness [19]. This framework is an updated version of McCormack and McCance’s [20] Person-centered Nursing Framework for applicability to a wide range of healthcare professionals, taking into account a multidisciplinary setting and interprofessional collaboration for person-centered practices. The PCPF comprises the four main domains: *prerequisites* focused on the staff` attributes which enable effective person-centered care, *practice environment* focused on the context in which healthcare is provided, *person-centered processes* with person-centered care delivered through various activities, and effective *person-centered outcomes* [19]. Person-centered care processes are influenced by both personal factors (prerequisites) and care environment factors, ultimately leading to effective person-centered outcomes. Personal factors include attributes of the health professionals, such as professionally competent (e.g., knowledge, attitudes, and skills), interpersonal skills, and job commitment [21, 22]. Similarly, care environment factors play a significant role in promoting or reinforcing person-centered care processes. These factors include supportive organizational systems [22], teamwork [23], and the physical work environment [24].

The well-being of residents in long-term care facilities is greatly dependent on the care provided by the staff, considering their cognitive and physical dependencies. Despite their reduced ability to express their needs and preferences, it is important to acknowledge that these residents still possess individual needs and preferences. Therefore, individualized care provided through SDM becomes essential. This study aims to determine the current status and influencing factors of SDM among long-term care staff including nursing staff, social workers, and personal care workers.

## Methods

### Study design

This study utilized a descriptive survey design to examine the current status of SDM among staff members at long-term care facilities. It also aimed to identify the personal characteristics of care providers and care environment characteristics that influence SDM, based on a PCPF.

### Study participants

The participants in this study included nursing staff (e.g., nurses and nurse aides), social workers, and personal care workers from 13 long-term care facilities in the Republic of Korea, selected through convenience sampling. The sample size was determined using the G-power program version 3.1.9.4 (Dusseldorf University, Dusseldorf, Germany), with a minimum sample size of 172 participants required for a multiple linear regression analysis considering 10 explanatory variables, an effect size of 0.15, an α error probability of 0.05, and a power of 0.95. Considering the possibility of missing data, a total of 300 participants were enrolled in this study. With the exclusion of 20 participants with missing data on the dependent variable (SDM), the data of 280 participants were analyzed.

### Conceptual framework

**Fig. 1:**
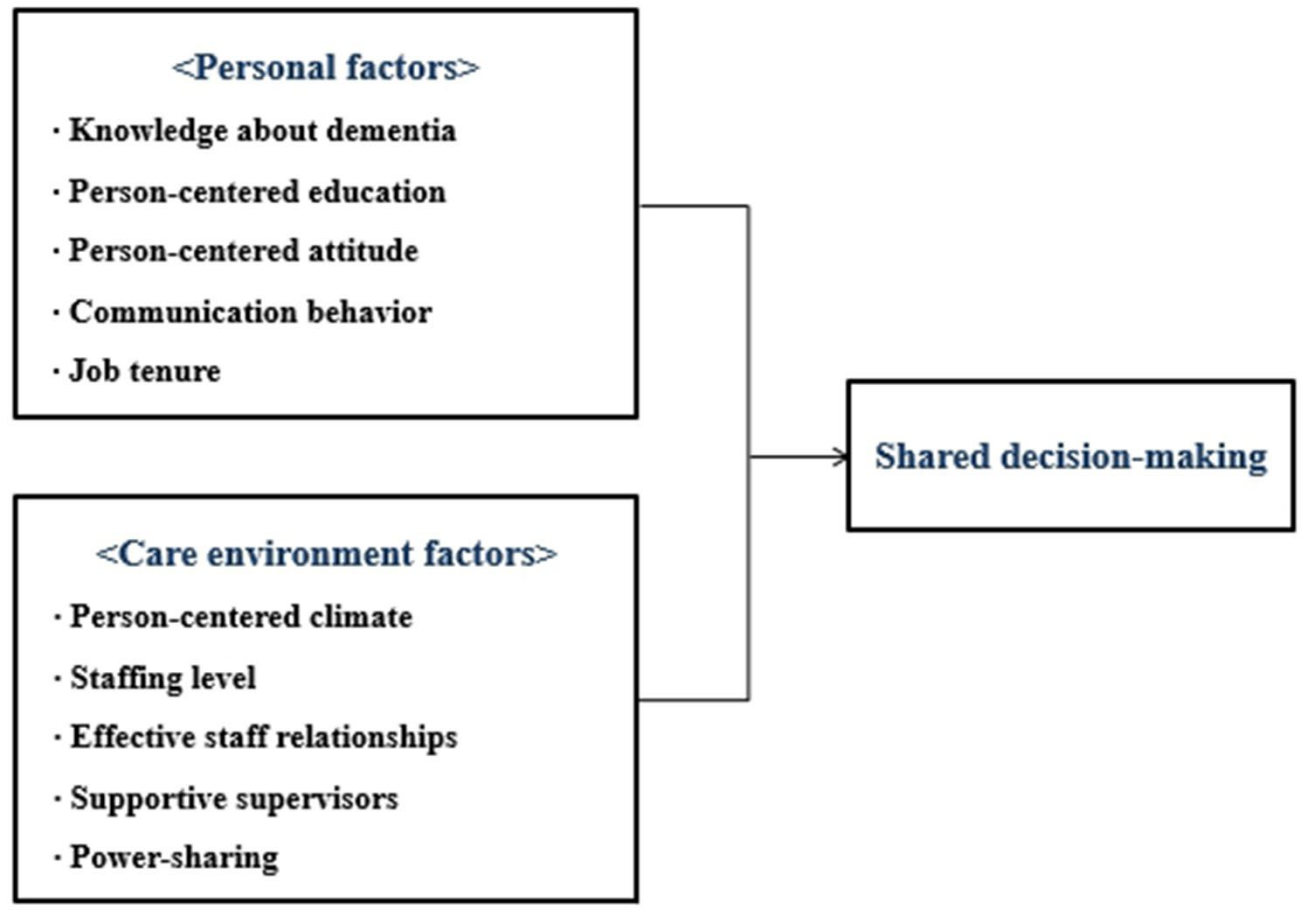
Conceptual framework of this study

The study employed the PCPF which outlines the factors influencing person-centered processes and outcomes [19]. According to this framework, person-centered nursing processes, including SDM, can be influenced by prerequisites and the practice environment. Prerequisites refer to the attributes of staff, such as professional competence (e.g., knowledge, attitudes, and skills), interpersonal and communication skills, and commitment to the job. The practice environment focuses on the environmental context where care is experienced, such as appropriate skill mix, effective staff relationships, supportive organizational systems, and power-sharing. The conceptual framework of this study was developed to identify the factors influencing SDM in long-term care facilities (Fig. 1). The personal factors corresponding to the prerequisites consist of knowledge about dementia, experience of person-centered care education, person-centered attitude, communication behavior, and job tenure. In addition, the care environment factors consist of a person-centered climate, staffing level, effective staff relationships, supportive supervisors, and power-sharing.

### Measurements

#### Shared decision-making (SDM)

To measure SDM, the Shared Decision Making Questionnaire - Physician version (SDM-Q-Doc), a tool developed by Scholl et al. [25] and subsequently translated into Korean by Park et al. [26], was used in this study after modifications were made to adapt it to the context of long-term care facilities, with permission from the original authors. For instance, terms such as “treatment” and “patient” were changed to “care” and “*Daesangja* (care receiver in Korean) or family,” respectively. The revised instrument was reviewed for its suitability for long-term care facilities by a nursing professor with expertise in person-centered care and a director of a long-term care facility. The instrument consists of nine items, each rated on a six-point Likert scale (from 0 “Completely disagree” to 5 “Completely agree”). The total score is obtained as the sum of all item scores, with higher total scores indicating higher levels of SDM. To assess the construct validity of the revised instrument, its correlation with the average score for person-centered care, as measured by the Person-centered Care Assessment Tool (P-CAT) [27], was analyzed (r = 0.568, *p* < 0.001). Furthermore, the instrument demonstrated high reliability in measuring SDM, with a Cronbach’s α coefficient of 0.950 in this study.

#### Personal factors

To measure the knowledge about dementia, this study employed a tool developed by Hwang & Jang [28], specifically designed for dementia care workers. The tool consists of 15 items categorized into two subscales (knowledge about dementia and knowledge about dementia care). Each item is rated as either correct (1 point) or incorrect (0 points), with higher scores indicating higher levels of dementia-related knowledge. For evaluating person-centered attitude, the Personhood in Dementia Questionnaire (PDQ) developed by Hunter et al. [29] and subsequently translated into Korean and validated by Kim et al. [30] was used. The tool consists of 20 items divided into three subscales: agency, respect for personhood, and psychosocial engagement, with each item rated on a seven-point Likert scale (from 1 for “Completely disagree” to 7 for “Completely agree”). Higher mean scores indicate a higher level of person-centered attitude among staff members. Internal consistency reliability for the PDQ showed Cronbach’s α = 0.873 in this study. For the assessment of communication behavior, the Communication Behavior Scale for nurses caring for people with Dementia (CBS-D) developed by Lee et al. [31] was used. The tool consists of 18 items on a scale from 1 for “Never” to 5 for “Always,” with higher mean scores indicating higher levels of appropriate communication behavior when interacting with patients with dementia. The Cronbach’s alphas for the CBS-D was 0.910 in this study. Job tenure was measured as the number of months that the staff had worked at the current long-term care facility.

#### Care environment factors

To measure person-centered climate, the Person-centered Climate Questionnaire-Staff (PCQ-S) developed by Edvardsson et al. [32] and subsequently translated into Korean and validated by Sagong et al. [33] was used. The tool consists of 17 items categorized into three subscales: safety, everydayness and community. Each item is rated on a six-point Likert scale (from 1 for “Completely disagree” to 6 for “Completely agree”), with higher mean scores indicating higher levels of person-centeredness within the care environment. Internal consistency reliability for the PCQ-S showed Cronbach’s α = 0.937 in this study. Regarding staffing level, the resident-to-personal care worker ratio was used as a measure. Additionally, the presence of effective staff relationships and supportive supervisors was assessed using a five-point Likert scale based on two items derived from the 6th Work Environment Survey conducted by the Korea Occupational Safety and Health Agency (KOSHA): “The cooperation between me and my coworker is good” and “My supervisor is helpful and supportive.” For evaluating power-sharing, a five-point Likert scale (1: Strongly disagree, 2: Disagree, 3: Neither agree nor disagree, 4: Agree, and 5: Strongly agree) was employed, based on the item “At the facility where I work, the decisions can be revised according to the suggestions made by the subordinate staff.”

### General characteristics of institutions and participants

The general characteristics of participating facilities included location (urban or rural), accreditation grade, operating periods, number of beds, and number of study participants. In terms of accreditation grade, the Korean National Health Insurance Service’s publicly recognized quality ratings in 2021 were used. The accrediting grade comprises five levels: A (superior), B (excellent), C (good), D (fair), and E (poor). Participants’ general characteristics included age, gender, educational attainment, marital status, religion, occupation type, and monthly salary.

### Data collection and ethical considerations

This study was approved by the Institutional Review Board at the author’s affiliated center (IRB No. 2022 − 0164). The study purpose and procedures were communicated to the directors or managers of the long-term care facilities. For the facilities that permitted the recruitment of study participants, the study purpose and procedures were then explained in detail to the nurses, nurse aides, personal care workers, and social workers working at those facilities. Participation in the study was entirely voluntary and only those who agreed to participate by providing their written consent were included. In addition, participants were informed that they could withdraw from the study if they so desired.

### Statistical analysis

Descriptive statistics were utilized to calculate the mean, standard deviation, percentage, and range of the data. Based on the median value of SDM, the participants were divided into two groups. Between-group differences were compared using χ^2^ tests or t-tests. Only variables with significant results in these difference tests were included in the regression analyses to determine the factors that influence SDM. Considering that statistical independence could be reduced due to workers within the same facility providing responses, the intra-cluster correlation coefficient (ICC) was calculated. As the ICC for SDM was determined to be 0.162, multilevel linear regression analyses were performed using Mplus Version 8.8. Given that SDM is an ordinal variable, a statistical adjustment was made using weighted least square mean and variance (WLSMV) estimation with robust standard errors.

## Results

### General characteristics of the long-term care facilities

Of the 13 long-term care facilities included in the sample, eight were located in urban areas and five were located in rural areas (Table 1). Nine facilities received a Grade A accreditation-quality rating, three received a Grade B rating, and one received a Grade C rating. The mean operation period was 11.92 ± 2.69 years, the mean number of beds was 53.85 ± 34.18, and the mean number of study participants per facility was 21.54 ± 10.88. Each institution’s descriptive statistics are displayed in further detail [see Additional file 1].

**Table 1:** General characteristics of the long-term care facilities (N = 13)

Variables	Categories	N (%) or Mean ± SD
Location	Urban	8 (61.5)
	Rural	5 (38.5)
Accreditation grade	Grade A (Superior)	9 (69.2)
	Grade B (Excellent)	3 (23.1)
	Grade C (Good)	1 (7.7)
Operating period (years)		11.92 ± 2.69
Number of beds		53.85 ± 34.18
Number of study participants		21.54 ± 10.88

### General characteristics of the participants

The mean age of the study participants was 53.41 ± 9.04 years (Table 2). The majority of participants were female (n = 250, 89.6%). Regarding educational attainment, the number of high school graduates was the highest (n = 146, 52.7%), followed by those with a college degree (n = 65, 23.5%). Regarding marital status, 219 participants (78.5%) were married, and 117 (42.5%) reported having no religious affiliation. Regarding occupation type, the number of personal care workers was the highest (n = 213, 76.3%), followed by social workers (n = 34, 12.2%), and nurses or nurse aides (n = 32, 11.5%). The mean monthly income was 2,002,500 ± 272,200 KRW.

**Table 2:** General characteristics of the study participants (N = 280)

Variables	Categories	N (%) or Mean ± SD
Age (years)		53.41 ± 9.04
Gender	Male	29 (10.4)
	Female	250 (89.6)
Educational attainment	Middle school or less	34 (12.3)
	High school	146 (52.7)
	College diploma	65 (23.5)
	Bachelor’s degree or more	32 (11.5)
Marital status	Without spouse	60 (21.5)
	With spouse	219 (78.5)
Religion	Yes	158 (57.5)
	No	117 (42.5)
Type of occupation	Registered nurse or nurse aid	32 (11.5)
	Social worker	34 (12.2)
	Personal care worker	213 (76.3)
Monthly salary (10,000 won)		200.25 ± 27.22

Number of missing data: age = 4, gender = 1, educational attainment = 3, marital status = 1, religion = 5, type of occupation = 1, monthly salary = 7

### Personal and care environment characteristics of the participants

For personal characteristics, the mean scores were as follows: 11.88 ± 2.32 for knowledge about dementia, 5.24 ± 0.86 for person-centered attitude toward residents with dementia, and 4.14 ± 0.52 for communication behavior (Table 3). A total of 169 participants (63.8%) had experience of person-centered care education. The average job tenure at the current facility was 55.58 ± 47.14 months. Regarding care environment characteristics, the mean score for person-centered climate was 5.08 ± 0.65 and the mean staffing level, which represents the current ratio of residents per personal care worker, was 2.25 ± 0.14. The mean score for effective staff relationships was relatively high at 4.27 ± 0.67, while the mean scores of supportive supervisors and power-sharing were 4.20 ± 0.77 and 3.69 ± 0.95, respectively. Each institution’s descriptive analysis of care environment factors is given in further detail [see Additional file 1].

**Table 3:** Personal and care environment characteristics by level of shared decision-making

Content	Variables	Total (N = 280)	Level of SDM		X^2^ or t	*p-value*
			High level of SDM group (n = 144) ^a^	Low level of SDM group (n = 136)		
		N (%) or Mean ± SD	N(%) or Mean ± SD	N (%) or Mean ± SD		
Personal factors	Knowledge about dementia	11.88 ± 2.32	12.10 ± 2.24	11.65 ± 2.39	-1.63	0.105
	Person-centered care education (ref. no)	169 (63.8)	97 (71.3)	72 (55.8)	6.89	**0.009**
	Person-centered attitude (PDQ)	5.24 ± 0.86	5.61 ± 0.82	4.85 ± 0.73	-8.18	**< 0.001**
	Communication behavior (CBS-D)	4.14 ± 0.52	4.36 ± 0.50	3.91 ± 0.43	-7.98	**< 0.001**
	Job tenure (months)	55.58 ± 47.14	61.37 ± 48.72	49.39 ± 44.76	-2.09	**0.038**
Care environment factors	Person-centered climate (PCQ-S)	5.08 ± 0.65	5.40 ± 0.48	4.74 ± 0.63	-9.82	**< 0.001**
	Staffing level (Resident-to-personal care worker ratio)	2.25 ± 0.14	2.25 ± 0.14	2.26 ± 0.14	0.63	0.527
	Effective staff relationships	4.27 ± 0.67	4.44 ± 0.65	4.08 ± 0.65	-4.71	**< 0.001**
	Supportive supervisors	4.20 ± 0.77	4.50 ± 0.63	3.89 ± 0.79	-7.21	**< 0.001**
	Power-sharing	3.69 ± 0.95	4.02 ± 0.92	3.34 ± 0.85	-6.43	**< 0.001**

*CBS-D* Communication Behavior Scale for nurses caring for people with Dementia, *PCQ-S* Person-centered Climate Questionnaire- staff version, *PDQ* Personhood in Dementia Questionnaire, *SD* standard deviation, *SDM* shared decision-making.

Number of missing data: person-centered care education = 15, communication behavior = 10, job tenure = 13.

Bold indicates statistical significance.

^a^ Total respondents were classified into two groups (high level vs. low level) according to the median value of the revised Shared Decision Making Questionnaire-Physician version (SDM-Q-Doc) score (37.0).

### Shared decision-making of the participants

The mean total score for SDM was 35.78 ± 8.19 (Table 4). The item with the highest score was “5. I helped my care receiver (or family) understand all the information.” (mean score = 4.14), and the item with the second-highest score was “3. I told my care receiver (or family) that there are different care options for his/her medical condition” (mean score = 4.10). The item with the lowest score was “7. My care receiver (or family) and I thoroughly weighed the different care options” (mean score = 3.75), and the item with the second-lowest score was “8. My care receiver (or family) and I selected a care option together.” (mean score = 3.83).

**Table 4:** Descriptive statistics of shared decision-making (N = 280)

Items	Mean ± SD	Min - Max
1. I made clear to my care receiver (or family) that a decision needs to be made.	4.09 ± 1.01	1–5
2. I wanted to know exactly from my care receiver (or family) how he/she wants to be involved in making the decision.	3.93 ± 1.04	0–5
3. I told my care receiver (or family) that there are different options for caring his/her medical condition.	4.10 ± 1.03	0–5
4. I precisely explained the advantages and disadvantages of the care options to my care receiver (or family).	4.04 ± 1.12	0–5
5. I helped my care receiver (or family) understand all the information.	4.14 ± 1.02	0–5
6. I asked my care receiver (or family) which care option he/she prefers.	3.98 ± 1.06	0–5
7. My care receiver (or family) and I thoroughly weighed the different care options.	3.75 ± 1.09	0–5
8. My care receiver (or family) and I selected a care option together.	3.83 ± 1.16	0–5
9. My care receiver (or family) and I reached an agreement on how to proceed.	3.92 ± 1.17	0–5
Total score	35.78 ± 8.19	8–45

### Comparison of personal, and care environment characteristics by level of shared decision-making

The total study participants were classified into two groups (high-level and low-level) according to the median value of the SDM score (median value = 37). In terms of personal characteristics, the high-level SDM group had a significantly higher percentage of individuals with experience in person-centered care education (χ2 = 6.89, *p* = 0.009; Table 3). Furthermore, the high-level SDM group outperformed the low-level SDM group in terms of person-centered attitude (t=-8.18, *p* < 0.001), communication behavior (t=-7.98, *p* < 0.001), and job tenure (t=-2.09, *p* = 0.038). Regarding care environment characteristics, the high-level SDM group exhibited higher levels of person-centered climate (t=-9.82, *p* < 0.001), effective staff relationships (t=-4.71, *p* < 0.001), supportive supervisors (t=-7.21, *p* < 0.001), and power-sharing (t=-6.43, *p* < 0.001) in long-term care facilities compared to the low-level SDM group.

### Regression analysis

Table 5 presents the results of multiple linear regression analyses. Among the personal factors, long-term care facility staff with experience of person-centered care education (β = 0.198, *p* = 0.034), a more person-centered attitude (β = 0.201, *p* = 0.007) and more appropriate communication behavior (β = 0.242, *p* < 0.001) were more likely to report a higher SDM score. Additionally, among the care environment factors, working at a facility with a more person-centered climate (β = 0.416, *p* < 0.001) was associated with a higher SDM score.

**Table 5:** Results of multiple linear regression analyses (N = 280)

Factors	Variables	Model 1 ^a^		Model 2 ^b^	
		β (SE)	*p*	β (SE)	*p*
Personal factors	Person-centered care education (ref. no)	-0.008 (0.048)	0.867	0.198 (0.093)	**0.034**
	Person-centered attitude (PDQ)	0.317 (0.073)	**< 0.001**	0.201 (0.074)	**0.007**
	Communication behavior (CBS-D)	0.335 (0.076)	**< 0.001**	0.242 (0.068)	**< 0.001**
	Job tenure (months)	0.122 (0.053)	**0.020**	0.069 (0.054)	0.195
Care environment factors	Person-centered climate (PCQ-S)			0.416 (0.100)	**< 0.001**
	Effective staff relationships			-0.008 (0.067)	0.910
	Supportive supervisors			0.089 (0.055)	0.104
	Power-sharing			-0.101 (0.065)	0.123
R square		0.334		0.435	

*β* standardized coefficient, *CBS-D* Communication Behavior Scale for nurses caring for people with Dementia, *PCQ-S* Person-centered Climate Questionnaire- staff version, *PDQ* Personhood in Dementia Questionnaire, *SE* standard error.

Bold indicates statistical significance.

^a^ Including personal factors.

^b^ Including personal and care environment factors.

## Discussion

This study examined the current status of SDM and identified the personal characteristics of care providers and the care environment characteristics that influence SDM among long-term care facility staff. The study employed a PCPF and identified the experience of person-centered care education, person-centered attitude, communication behavior, and person-centered climate as key factors influencing SDM.

The mean score of SDM in this study was 35.78, which was slightly higher than the mean score observed among physicians in primary care clinics in Japan (31.65) using the same measurement tool [34]. This difference could be attributed to the increased emphasis on reflecting the values and preferences of residents in long-term care facilities, where care services are closely related to the daily activities of residents, such as having a meal and urinating. In another study conducted in Japan using the same instrument among a sample of home care staff, the mean scores for Items 4 and 7 were the lowest [35]. These findings align with the present study, where the mean scores of Items 7 and 8 were the lowest. Notably, the score for Item 7 (My care receiver (or family) and I thoroughly weighed the different care options) was low in both studies. This implies that despite engaging in discussions regarding care options with the care receiver or family, various care methods are not being comparatively assessed adequately. Furthermore, the score of Item 8 (My care receiver (or family) and I selected a care option together) was low in the current study, indicating that the final decision regarding the care option is highly likely to be made by the care provider. Hence, it is crucial to develop effective strategies that promote increased engagement of the care receiver or family in selecting care options.

Regarding the personal characteristics analyzed in this study, the experience of person-centered care education was found to have a significant impact on SDM. SDM is a communication approach based on the philosophy of person-centered care, which emphasizes incorporating the preferences of the care receiver, addressing their physical, psychological, and social needs, and promoting their engagement [13, 36]. Therefore, education on person-centered care could have a positive impact on the practice of SDM. Nevertheless, it is worth noting that in this study, only 63.8% of participants had experienced person-centered care education, and approximately one-third had no experience in this area. It is thus necessary to reinforce person-centered care education for staff in long-term care facilities to promote SDM.

In addition, a person-centered attitude was found to significantly influence SDM. Person-centered attitude encompasses the care receiver’s sense of agency, belief in their competency for psychosocial engagement, and respect for personhood [30]. Of these, attitude toward agency, in particular, can have a significant impact on SDM as it reflects the care provider’s belief in the care receiver’s ability to make decisions regarding their own actions. A study implementing a person-centered dementia care education program, which included curriculum components focused on “valuing people” and “personal perspectives” for the staff at nursing homes, demonstrated improvements in person-centered attitude [37]. Notably, it is necessary to improve person-centered attitudes by providing person-centered care education through various approaches such as incorporating videos or images [37], ICT technologies such as touchscreen technology [38], and role-playing strategies [39], subsequently enhancing SDM practices.

Furthermore, effective communication behavior in care providers involves considering and accommodating the care receiver’s communication impairments and needs [31]. By engaging in appropriate communication behavior, care providers can gain a better understanding of the values and preferences of care receivers and their families, facilitating the expression of opinions and promoting effective interaction. In addition, demonstrating empathy can enhance the interaction between care providers and care receivers. In a previous study, as the person-centered communication by the staff increased, the positive reactions of residents at care facilities increased [40]. Hence, it is crucial to provide educational programs for care providers that focus on improving their communication skills [41].

Especially, as most residents in long-term care facilities may experience cognitive decline and have difficulty expressing their opinions, it is necessary to develop tools or strategies to assist in their communication. These tools can bridge the communication gap between care receivers and care providers and encourage family participation. Cranley et al. have recently developed a communication tool specifically designed for interacting with individuals with dementia, suggesting that it can be utilized in a structured format known as SBARR (Situation, Background, Assessment, Recommendation, and Read back or Response) during communication [42]. Employing a step-by-step communication approach guided by such tools following a guideline may facilitate meaningful discussions about care and provide support to residents in care facilities as they engage in the decision-making process.

In terms of the care environment characteristics, the results of this study confirmed that staff working at care facilities with a higher level of person-centered climate reported a higher level of SDM. The score for person-centered climate in this study was slightly higher than the mean score (4.82; SD = 0.55) of the staff at long-term care facilities obtained during the development of the Korean version of the PCQ-S [33]. The components of the person-centered climate include safety, everydayness and community, whereby the residents at care facilities feel at home and enjoy an encouraging environment for building harmonious relationships with the staff and other residents [33]. Person-centered care is often referred to as a culture-change movement, where transforming the environment, including the overall organizational culture, enables facilities to provide person-centered services [43]. It is noteworthy that the nurse-patient interaction is more strongly influenced by organizational culture than the individual competence of the nurse [44]. Therefore, directors and managers of care facilities should make efforts to foster a person-centered environment for staff to provide better person-centered services, including SDM.

Although the present study reveals important findings, it has several limitations. First, as the study participants were recruited from only 13 long-term care facilities, it might be limited to generalize these results. In the future, large-scale studies should be conducted using a more representative sample of staff from various long-term care facilities. Second, as a cross-sectional study, the cause-and-effect relationship between SDM and the explanatory variables of this study could not be verified. Thus, a prospective longitudinal study should be conducted as a follow-up to examine the causal relationship between these variables.

## Conclusion

SDM involves long-term interactions between care receivers or their families and care providers, encompassing information exchange, discussions, and reaching mutual agreements aimed at providing individualized care that reflects the values, preferences, and functional levels of the care receiver. The results of this study have identified several key factors that influence SDM in long-term care facilities, including the experience of person-centered care education, person-centered attitude, communication behavior, and person-centered climate. These findings are anticipated to provide practical insights to promote SDM within long-term care facilities, ultimately contributing to enhanced quality of care and the improved quality of life for residents.

### Electronic supplementary material

Below is the link to the electronic supplementary material.

Supplementary Material 1

## Data Availability

The datasets generated and/or analysed during the current study are not publicly available, but are available from the corresponding author on reasonable request.

## Abbreviations

SDM: Shared decision-making
PCPF: Person-centered Practice Framework
SDM-Q-Doc: Shared Decision Making Questionnaire - Physician version
P-CAT: Person-centered Care Assessment Tool
PDQ: Personhood in Dementia Questionnaire
CBS-D: Communication Behavior Scale for nurses caring for people with Dementia
PCQ-S: Person-centered Climate Questionnaire-Staff
KOSHA: Korea Occupational Safety and Health Agency
ICC: Intra-cluster Correlation Coefficient
WLSMV: Weighted Least Square Mean and Variance
SBARR: Situation, Background, Assessment, Recommendation, and Read back or Response
