# Supplementary material for: Factors influencing shared decision-making in long-term care facilities
Source: BMC Geriatr. 2023 Sep 19;23:577. doi: 10.1186/s12877-023-04301-6 (PMC10508015; doi:10.1186/s12877-023-04301-6)
Supplement: Supplementary file 1 — Supplementary Material 1 [file 12877_2023_4301_MOESM1_ESM.pdf]

## Additional file 1. General and care environment characteristics by each institution

| **Variables** | **Long-term care facilities** | | | | | | | | | | | | |
|---|---|---|---|---|---|---|---|---|---|---|---|---|---|
| | **A** | **B** | **C** | **D** | **E** | **F** | **G** | **H** | **I** | **J** | **K** | **L** | **M** |
| | **Value or Mean**±SD | | | | | | | | | | | | |
| ***General characteristics*** | | | | | | | | | | | | | |
| Location | Urban | Urban | Urban | Urban | Urban | Urban | Urban | Urban | Rural | Rural | Rural | Rural | Rural |
| Accreditation grade | A | A | A | A | A | A | A | B | A | C | B | B | A |
| Operating period (years) | 12 | 14 | 8 | 14 | 14 | 9 | 14 | 14 | 9 | 7 | 14 | 14 | 12 |
| Number of beds | 36 | 27 | 56 | 141 | 87 | 35 | 49 | 27 | 44 | 26 | 93 | 53 | 26 |
| Number of study participants | 20 | 15 | 23 | 55 | 28 | 21 | 15 | 13 | 20 | 15 | 21 | 19 | 15 |
| ***Care environment characteristics*** | | | | | | | | | | | | | |
| Person-centered climate (PCQ-S) | 4.90±0.40 | 5.20±0.64 | 5.19±0.57 | 5.04±0.58 | 5.05±0.63 | 5.34±0.63 | 4.72±0.46 | 5.64±0.49 | 4.69±0.87 | 5.33±0.62 | 5.25±0.55 | 5.33±0.60 | 4.51±0.67 |
| Staffing level (Resident-to-personal care worker ratio) | 2.12 | 2.08 | 2.43 | 2.20 | 2.12 | 2.06 | 2.13 | 2.45 | 2.44 | 2.36 | 2.27 | 2.41 | 2.36 |
| Effective staff relationships | 4.50±0.61 | 4.67±0.49 | 4.39±0.66 | 4.15±0.59 | 4.11±0.88 | 4.57±0.60 | 4.13±0.35 | 4.69±0.63 | 3.85±0.75 | 4.00±0.85 | 4.33±0.48 | 4.42±0.61 | 4.00±0.54 |
| Supportive supervisors | 4.35±0.49 | 4.47±0.52 | 4.09±0.95 | 3.96±0.77 | 4.39±0.96 | 4.76±0.54 | 3.93±0.59 | 4.69±0.63 | 3.75±0.85 | 4.13±0.74 | 4.29±0.64 | 4.37±0.68 | 3.87±0.52 |
| Power-sharing | 3.70±0.66 | 4.00±0.66 | 3.61±1.08 | 3.42±0.96 | 3.57±0.84 | 4.05±0.87 | 3.07±1.03 | 4.62±0.87 | 3.20±0.95 | 3.60±1.18 | 4.19±0.75 | 4.05±0.91 | 3.60±0.51 |

*PCQ-S* Person-centered Climate Questionnaire-staff version, *SD* standard deviation
Accreditation grade A means superior, B means excellent, and C means good.
